# Leaf Physiological Plasticity in *Schima superba* and *Schima argentea* is Related to Ecological Niche Width Under Varied Altitude Gradients

**DOI:** 10.1002/ece3.71793

**Published:** 2025-07-14

**Authors:** Deng Wang, Yeshe Wang, Zhezhi Li, Shu Wang, Lijun Chen

**Affiliations:** ^1^ College of Agriculture, Forestry and Ecology Shaoyang University Shaoyang China; ^2^ College of Forestry, Forest Ecology Research Center Guizhou University Guiyang China

**Keywords:** morphological plasticity, Nanshan National Park, physiological plasticity, *Schima*

## Abstract

Plasticity magnitude may affect the distribution and adaptability of species in altitude gradients. The term is broadly defined as the adaptability of organisms to alter their morphological and physiological traits in response to varying environments. Morphological and physiological plasticity may have different mechanisms and resource costs. However, our understanding of the mechanisms by which plasticity affects species' adaptation to altitude changes is limited. This study focused on the differences in the leaf traits of *Schima superba* (narrow ecological niche) and *S. argentea* (wider ecological niche) in response to altitude gradients. It also explored the adaptive strategies and mechanisms behind the plasticity of morphological and physiological traits under similar environmental pressures. The interaction between altitude and species significantly impacted morphological traits, such as leaf thickness, width, and mass, and physiological traits, such as chlorophyll, carotenoids (Car), relative water, soluble sugar (SS), leaf nitrogen (LNC), and leaf phosphorus (LPC) contents, as well as the N/P ratio. The leaf traits of the two species responded similarly to altitude gradient changes, but the adaptive potential of *S. argentea* was higher. Compared with *S. superba* , the chlorophyll content of *S. argentea* at high altitude (1912 m) was remarkably greater than that at two lower altitudes (1375 and 1552 m). Moreover, it was affected by nitrogen and phosphorus limitation only when the altitude exceeded 1912 m. Quantitative analysis based on the simplified relative distance plasticity index (RDPI_s_) showed that the RDPI_s_ of physiological traits in *S. argentea* were significantly greater than those of morphological traits, and the RDPI_s_ of most physiological traits were greater than those of *S. superba* , mainly due to the RDPI_s_ of its physiological traits—especially LNC (0.357), Car (0.328), and SS (0.319). Thus, physiological plasticity plays a critical role in adapting to environmental changes, especially in the case of vertical gradients.

## Graphical abstract

Leaf traits of two *Schima* species differed significantly with altitude changes. *Schima argentea* had higher physiological than morphological plasticity versus *S. superba*. Ecological niche width positively correlated with plasticity of leaf physiological traits.

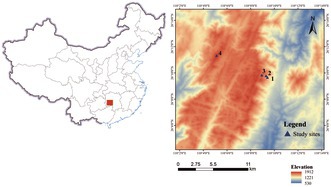

## 1 Introduction

As an important and comprehensive geographical factor, altitude gradient demonstrates wide variability in environmental factors such as temperature, humidity, UV radiation, light, rainfall, and soil fertility, resulting in drastic climate changes within a narrow gradient range (Xu et al. 2017). In addition to harsher climatic conditions, an increase in elevation is characterized by a decline in the availability of resources, shorter growth seasons, reduced microbial activity, and low human population densities (Ahmad et al. 2019). Therefore, the performance of plants located at the edges of the population altitudinal distribution could significantly differ from those located at intermediate altitudes that experience a more favorable environment (Boscutti et al. 2018). In this context, the overall phenotypic plasticity of a plant species plays critical roles in the acclimation and adaptation processes along environmental gradients, including altitude (Rivas‐Ubach et al. 2025). Phenotypic plasticity, the phenomenon or ability of organisms to produce different phenotypes under varied environments, allows flexible and rapid morphological, physiological, and biochemical responses to environmental changes (Pfennigwerth et al. 2017; Rivas‐Ubach et al. 2025). Exploring the plasticity of plants is a prerequisite for understanding the short‐term adaptive responses of different species to environmental changes (Pfennigwerth et al. 2017). Plasticity may play a crucial role in plants' responses to climate change and enable their establishment and persistence in varied geographical or altitudinal ranges (Gratani et al. 2014; Bakhtiari et al. 2019). Genetic responses may also promote adaptation, although not as quickly as plasticity (Liao et al. 2019).

The leaf is the plant organ with the greatest contact with the external environment; it is the most sensitive to environmental changes; and it directly participates in photosynthetic carbon fixation, respiration, and transpiration (Carlson et al. 2015; Bakhtiari et al. 2019). Leaf functional traits can objectively reflect the environmental adaptability of plants. Common ones include leaf size, leaf thickness, leaf area, leaf mass, specific leaf area, and other morphological structural traits. Plasticity of these traits is an effective means for plants to respond appropriately to long‐term climate change (Guo et al. 2017). Leaf length, leaf width, leaf area, leaf mass, and specific leaf area significantly decreased, while leaf thickness markedly increased with elevation (Montti et al. 2014; Liu et al. 2020; Zhang et al. 2020; Siraj et al. 2023), and low temperatures and a strong radiation environment at high altitudes enhance the survival pressure on plants, forcing them to reduce transpiration rate, prolong life cycle, and slow down growth by reducing leaf area, leaf mass, and specific leaf area but increasing leaf thickness, thus forming a conservative, resource utilization strategy (Lin et al. 2021; Kramp et al. 2022; Yu et al. 2022). Changes in leaf photosynthetic pigments, nutrient levels, water use efficiency, osmotic compounds, metabolites, and other physiological traits can reveal the mechanisms by which plants optimize their resource allocation strategies to maximize survival and reproduction under different environments (Sardans et al. 2020; Bin et al. 2022; Rivas‐Ubach et al. 2025). Low temperature and enhanced radiation at high altitudes reduce the metabolic rates of trees and the demand for nitrogen and phosphorus, elevating the leaf nitrogen and phosphorus contents (Michaletz 2018; Bin et al. 2022). These alterations can upregulate plant soluble sugar and leaf potassium contents to reduce reactive oxygen species‐induced damage to cell structure, regulate intracellular osmotic pressure, prevent cell dehydration, and enhance cold and stress resistance (Keunen et al. 2013; Xu et al. 2018; Rahman et al. 2020; Rivas‐Ubach et al. 2025). A thickened leaf cuticle can suppress the transpiration rate, preventing water loss and increasing the relative water content (Adamidis et al. 2021). Simultaneously, the enhanced carotenoid and reduced chlorophyll contents under high radiation conditions may be interpreted as an effective mechanism to avoid (Li et al. 2022). Therefore, plants can coordinate the plasticity of multiple leaf functional traits to optimize their resource utilization and allocation as well as physiological and ecological adaptation strategies under altitude gradient changes (Li et al. 2021; Wang et al. 2022; Bin et al. 2022; Liu et al. 2023).

Morphological and physiological plasticity is believed to have different mechanisms, resource costs, and ecological implications (Grime and Mackey 2002). Morphological plasticity requires a longer period and more resource investment to achieve higher survival competitiveness in different environments by changing organ structures and tissues. In contrast, physiological plasticity is short‐lived and occurs in differentiated tissues that are imperceptible to the naked eye, and helps plants grow and reproduce successfully in heterogeneous habitats by regulating internal physiological processes and functions (Sultan 2000; Wang et al. 2023). Therefore, it is generally believed that species with greater morphological and physiological plasticity may be better able to adapt to new or stressful environments, helping to expand their geographic or altitude range under different environmental conditions (Molina Montenegro et al. 2012; Hou et al. 2015; Pfennigwerth et al. 2017). However, plants may need to prioritize one type of plasticity to maximize their ecological niche width and competitiveness under specific environmental conditions. Therefore, understanding the trade‐off between morphological and physiological plasticity as well as their relative importance in different environments is crucial for revealing plant adaptation mechanisms and predicting species distributions.

*Schima superba* and *S. argentea* (Theaceae) are two dominant tree species widely distributed in subtropical, evergreen, broad‐leaved forests in China. According to the Flora of China, the shade tolerance of *S. argentea* is relatively low, and it can adapt to climate changes in a vertical gradient at altitudes between 1750 and 2700 m. The annual average temperature of its primary area of distribution is 11°C–15°C; in contrast, the suitable altitude range of *S. superba* is between 1200 and 1400 m, and the annual average temperature of its core distribution area is 15°C–22°C (Wu et al. 2023; Jiang et al. 2023). Therefore, compared with *S. superba* , *S. argentea* has a wider ecological range, which is manifested in its adaptability to ecological factors, such as altitude, light intensity, and temperature. Our field investigation revealed that a large area of native *S. superba* and *S. argentea* communities existed in an area extending from an altitude of 1000 to 1946 m in the Nanshan National Park, Hunan Province, China. This pattern provides an ideal platform for studying the plasticity of closely related species in different ecological ranges under altitude gradient changes and exploring their possible mechanisms and resource costs. We speculate that *S. argentea* may exhibit higher plasticity than *S. superba* , which enables it to survive at higher altitudes. To test this hypothesis, 16 traits were measured to determine their plasticity to variations in altitude gradients. The main objectives of this study were to determine the following: (1) how *S. superba* and *S. argentea* adapt to variation in altitude gradients in terms of morphology and physiological traits; and (2) whether *S. argentea* exhibits broader physiological or morphological plasticity than *S. superba* .

## 2 Materials and Methods

### 2.1 Study Area and Plant Material

Hunan Nanshan National Park (26°01′–26°21′ N, 109°59′–110°33′ E) is located at the Nanling Mountain peaks, at an intersection of the east–west and north–south mountains in China, across the evergreen broad‐leaved forests of the eco‐geographical regions of the hilly basin on the south bank of the Yangtze River and Wuling Mountain. The region is located in the central subtropical zone, with a subtropical and monsoon humid climate dominated by mountains. The terrain varies greatly in altitude (425–1946 m). It is successively divided into subtropical evergreen broad‐leaved, evergreen mixed deciduous broad‐leaved, deciduous broad‐leaved, and mountain‐top dwarf forest belts. The average annual temperature is 16.1°C, the annual sunshine duration is 1138 h, the frost‐free period is 271 days, the relative humidity is 75%–83%, the annual rainfall is 1100–1400 mm (mostly concentrated in May–June), and the soil types are yellow‐brown soil, which is classified as Podzoluvisols (according to the FAO soil classification).

**FIGURE 1:**
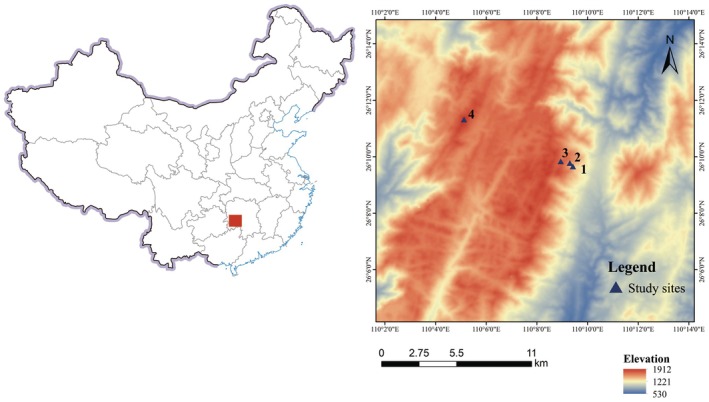
Map showing the study area and sampling plots.

Preliminary surveys were performed to identify sites dominated by *S. superba* and *S. argentea*. Four sample plots (50 × 50 m) were identified at elevation intervals of approximately 200 m located on the southern slopes of the Nanshan National Park along an elevation gradient ranging from 1375 to 1912 m. The elevations of the four plots were 1375, 1552, 1716, and 1912 m, respectively. *S. superba* and *S. argentea* were two plant species distributed in these plots; their heights ranged between 8.3–4.3 m and 9.5–5.2 m (Table 1). Samples were collected in July 2022. To minimize the influence of individual growth environment heterogeneity at the same altitude on the experimental results, 5 individuals were selected from each of the two populations of *S. superba* and *S. argentea* at each sampling site, and 10–20 fully expanded sun leaves were collected from each individual. The collected leaves of each individual were mixed thoroughly and evenly, then divided into marked plastic bags and placed in an incubator at 4°C for refrigeration. In total, leaves from eight populations were sampled, including four each of *S. superba* (1375–1912 m) and *S. argentea* (1375–1912 m; Figure 1). The trees used for sampling were ensured to be healthy, disease–free, and devoid of physical injury.

**TABLE 1:** Study site information of *Schima superba* and *S. argentea* .

Site name	Elevation (m)	Latitude and longitude	MS (°)	AMT (°C)	TAR (°C)	AP (mm)	Plant height (m)	
							*S. superba*	*S. argentea*
S1	1375	110°9′25′′ E, 26°9′39′′ N	28	11.99	27.08	1637	8.3 ± 1.1	9.5 ± 1.5
S2	1552	110°9′18′′ E, 26°9′47′′ N	30	11.97	27.00	1637	6.2 ± 1.0	7.3 ± 1.2
S3	1716	110°8′58′′ E, 26°9′52′′ N	38	11.20	26.90	1661	5.7 ± 0.8	6.8 ± 1.4
S4	1912	110°5′7′′ E, 26°11′18′′ N	43	11.05	26.80	1654	4.3 ± 0.5	5.2 ± 0.8

Abbreviations: AMT, annual mean temperature; AP, annual precipitation; MS, mean slope; TAR, temperature annual range.

### 2.2 Measurement of Morphological and Physiological Traits

From each individual, 3 leaf samples were randomly selected for the measurement of morphological traits (3 samples × 5 individuals × 4 altitude gradients × 2 species = 120 samples in total). The Yaxin‐1241 leaf area meter (Beijing Yaxinliyi Science and Technology Co. Ltd., Beijing, China) was used to measure leaf length (LL), leaf width (LW), and leaf area (LA). An electronic digital vernier caliper (accuracy 0.01 mm) was used to measure the leaf thickness (LT) while avoiding the main vein. The leaves were weighed using an electronic balance (accuracy 0.001 g) to obtain fresh mass. Then, each leaf was placed in an envelope bag and incubated in a 105°C oven for 30 min. After drying at 70°C to a constant weight was achieved, the leaf mass (LM) was determined. Finally, the specific leaf area (SLA) was calculated as the leaf area/leaf mass.

From each individual, 3 leaf samples were randomly selected for the measurement of physiological traits (3 samples × 5 individuals × 4 altitude gradients × 2 species = 120 samples in total). The relative water content (RWC) was determined using the conductance method (Gao 2006). Sampled leaves were weighed as fresh weight (W_1_), subsequently soaked in distilled water for 24 h in the dark to measure the saturated weight (W_2_), oven‐dried at 105°C for 15 min, and then dried to a constant weight (W_3_) at 80°C. The RWC was calculated by applying the following formula: RWC = (W_1_ − W_3_)/(W_2_ − W_3_).

To determine chlorophyll a (Chl a), chlorophyll b (Chl b), total chlorophyll (T Chl), and carotenoids (Car) contents, 10 mL of ice‐cold 80% (v/v) acetone was used to extract these pigments from 0.05 g of fresh leaves. After mixing overnight and centrifuging for 10 min at 12,000 rpm, the supernatant was collected, and its absorbance was measured at 663, 646, and 470 nm. The Chl and Car concentrations were calculated using the equations described by Lichtenthaler (1987).

Soluble sugar content (Ss) was determined using anthrone colorimetry (Li 2000). About 0.5 g of fresh leaves were ground in liquid N_2_. The solution was taken in a test tube, added with 5 mL of distilled water, and the SS was extracted by placing it in boiling water for 30 min. After the solution cooled down to 25°C, it was centrifuged at 5000 rpm for 5 min. The supernatant was poured into a 25 mL volumetric flask, and the extraction was repeated twice with constant volume. About 0.5 mL of the extract was taken in a hard test tube, and added with ~1.5 mL of distilled water, 0.5 mL of anthrone–ethyl acetate reagent, and 5 mL of concentrated sulfuric acid in an ice bath. After mixing, the solution was quickly heated in boiling water for 1 min, and the OD_630_ was measured.

The plant samples were dried for 72 h at 60°C and ground using an MM 400 ball mill (Retsch, Haan, Germany). AZ‐2300 NaOH melting‐flame atomic absorption spectrophotometer (Hitachi Company, Japan) was used to determine the leaf potassium content (LKC). Leaf nitrogen content (LNC) was determined using the Kjeldahl method, and leaf phosphorus content (LPC) employing the Mo‐Sb colorimetry (Gao et al. 2023). Finally, we calculated the N:P ratios (LN/P) to assess the N and P limitations of the plant.

### 2.3 Data Analysis

All data were analyzed using SPSS 18.0 software (IBM, NY, USA). Prior to statistical analysis, the Shapiro–Wilk test was applied to assess data normality and the Bartlett test was applied for homogeneity of variances. Tukey's HSD test following one‐way ANOVA was used to compare the differences in leaf morphology and physiological traits of each species at different altitude gradients. The final data presented are the means ± standard error. For morphology and physiological traits, two‐way ANOVA was conducted for effects of species, altitude gradients, and their interactions. A paired *t*‐test was applied to examine the difference in RDPI_s_ and average RDPI_s_ of morphological and physiological traits among species. The Origin 2020 software (https://www.originlab.com/) was used for data visualization.

Relative distances plasticity index (RDPI) was used to quantify the plasticity of individual traits for each species, with the following formula: RDPI = ∑ (*d*_*ij*→*i*′*j*′_/(*x*_*i*′*j*′_ + *x*_*ij*_))/*n*.

RDPI was computed as the Euclidean distances (*d*) between the trait values to different altitudes (*ij* and *i*′*j*′, respectively). To normalize the distances, the d value was divided by the sum of the absolute trait values (*x*_*i*′*j*′_ + *x*_*ij*_), where “*n*” is the total number of distances. When the number of replicates, species, and environments had excessively complicated calculations, the index was simplified (RDPI_s_) by calculating the distances among mean phenotypic values for each species–environment combination (Valladares et al. 2006).

## 3 Results

### 3.1 Morphological Trait Responses to Altitude Gradient Variations

**FIGURE 2:**
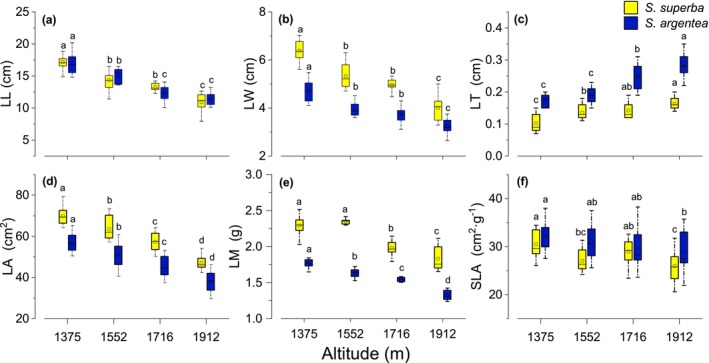
Altitudinal variation in leaf length (LL; a), leaf width (LW; b), leaf thickness (LT; c), leaf area (LA; d), leaf mass (LM; e), and specific leaf area (SLA; f) for *S. superba* and *S. argentea* . Data are presented as means ± SE. Different letters indicate significant differences among elevations based on ANOVA followed by Tukey's HSD test (*p* < 0.05).

The effects of altitude gradient and species on LW, LT, LA, LM, and SLA were significant, and the interaction between the altitude gradient and species was remarkable for LW, LT, and LM (*p* < 0.001; Table 2). For both species, LL, LW, LA, LM, and SLA decreased with increasing altitude, whereas LT showed the opposite trend (Figure 2a–f). Compared with *S. argentea* , the LT of *S. superba* showed minimal change at altitudes of 1552, 1716, and 1912 m (*p* < 0.001 vs. *p* = 0.848; *p* = 0.056; Figure 2c). No significant difference in LM was observed between 1375 and 1552 m (*p* < 0.001 vs. *p* = 0.580; Figure 2e), whereas SLA decreased markedly at 1912 m, showing a significantly lower value than that at 1716 m (*p* = 0.928 vs. *p* < 0.001; Figure 2f).

**TABLE 2:** *F*‐values from two‐way ANOVA for leaf length (LL), leaf width (LW), leaf thickness (LT), leaf area (LA), leaf mass (LM), and specific leaf area (SLA) for the effects of altitude gradient (AG), species (SP), and their interactions.

Source	Df	LT (cm)	LL (cm)	LW (cm)	LA (cm^2^)	LM (g)	SLA (cm^2^ · g^−1^)
AG	3	64.74***	139.77***	121.33***	99.19***	155.70***	8.65***
SP	1	343.76***	0.01	316.25***	172.00***	1056.14***	19.16***
AG × SP	3	11.02***	2.39	7.28***	0.85	11.41***	2.01

* *p* < 0.05.

** *p* < 0.01.

*** *p* < 0.001.

### 3.2 Physiological Trait Related to Altitude Gradient Variations

**FIGURE 3:**
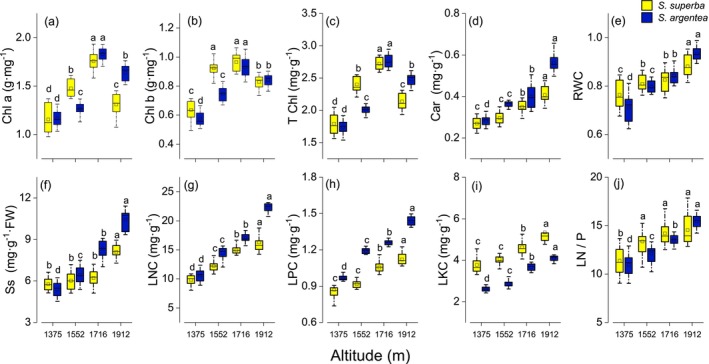
Altitudinal variation in chlorophyll a content (Chl a; a), chlorophyll b content (Chl b; b), total chlorophyll content (T‐Chl; c), carotenoid content (Car; d), relative water content (RWC; e), soluble sugar content (SS; f), leaf nitrogen content (LNC; g), leaf phosphorus content (LPC; h), leaf potassium content (LKC; i), and nitrogen‐to‐phosphorus ratio (LN/P; j) for *S. superba* and *S. argentea* . Data are presented as means ± SE. Different letters indicate significant differences among elevations based on ANOVA followed by Tukey's HSD test (*p* < 0.05).

Although species influenced all traits except RWC and LN/P (*p* < 0.01), the interaction between altitude gradient and species was remarkable for all traits except LKC (*p* < 0.01; Table 3). With increasing altitude, Chl a, Chl b, and T‐Chl in both species initially increased and then decreased, reaching their maximum values at 1716 m. Other traits showed a consistent upward trend with increasing altitude (Figure 3a–j). Compared with *S. argentea* , the contents of Chl a, Chl b, and T‐Chl in *S. superba* decreased significantly at 1912 m and were markedly lower than those at 1552 and 1716 m (*p* < 0.001; Figure 3a–c). No remarkable variation was observed in Car, LPC, and LKC between 1375 and 1552 m (*p* < 0.001 vs. *p* = 0.151, *p* = 0.208, and *p* = 0.264; Figure 3d,h,i). There were no significant differences in SS between 1375 and 1716 m (*p* < 0.001 vs. *p* = 0.139; Figure 3f), or in LN/P between 1552 and 1912 m (*p* < 0.001 vs. *p* = 0.098; Figure 3j).

**TABLE 3:** *F*‐values from two‐way ANOVA for chlorophyll a content (Chl a), chlorophyll b content (Chl b), total chlorophyll content (T Chl), carotenoid content (Car), relative water content (RWC), soluble sugar content (Ss), nitrogen content (LNC), phosphate content (LPC), potassium content (LKC), and leaf nitrogen/phosphate content (LN/P) for the effects of altitude gradient (AG), species (SP), and their interactions.

Source	df	Chl a (mg · g^−1^)	Chl b (mg · g^−1^)	T Chl (mg · g^−1^)	Car (mg · g^−1^)	RWC	SS (mg · g^−1^ · FW)	LNC (mg · g^−1^)	LPC (mg · g^−1^)	LKC (mg · g^−1^)	LN/P
AG	3	235.90***	207.02***	404.84***	187.67***	96.24***	217.78***	444.28***	286.94***	138.89***	62.63***
SP	1	8.45**	41.78***	33.25***	121.42***	0.03	105.41***	228.26***	608.72***	380.53***	2.90
AG × SP	3	41.72***	16.26***	257.67***	21.42***	9.33***	36.52***	37.41***	28.75***	1.21	4.82**

* *p* < 0.05.

** *p* < 0.01.

*** *p* < 0.001.

### 3.3 RDPI_s_ of Morphological and Physiological Traits in Response to Variation in Altitude Gradients

Except for LN/P, the RDPIs of other physiological traits in *S. argentea* were significantly higher than those in *S. superba* (*p* < 0.05); the RDPI_s_ values for LNC, Car, and SS were the highest in *S. argentea* (RDPI_s_ = 0.357, 0.328, and 0.319, respectively). The RDPI_s_ of LM in *S. argentea* was also significantly higher than that in *S. superba* (*p* = 0.048). *S. superba* demonstrated greater RDPI_s_ only in LW (*p* = 0.028; Table 4). The overall average RDPI_s_ of physiological traits in *S. argentea* was significantly higher than that of its morphological traits (RDPI_s_ = 0.226 vs. 0.140, *p* < 0.001), whereas the overall average RDPI_s_ of morphological and physiological traits in *S. superba* did not differ significantly. Although not statistically significant, the average RDPI_s_ of physiological traits in *S. argentea* was higher than that in *S. superba* .

**TABLE 4:** RDPI_s_ of leaf morphology and physiology traits of *S. superba* and *S. argentea* and the average RDPI_s_ of leaf morphology and physiology traits of the two species. Various symbols indicate significant differences among species based on paired *T*‐test (*p < 0.05; **p < 0.01; ***p < 0.001).

Traits	*S. superba*	*S. argentea*	Traits	*S. superba*	*S. argentea*
LL	0.226	0.198	T Chl	0.089	0.175***
LW	0.230*	0.181	Car	0.202	0.328***
LT	0.237	0.243	SS	0.168	0.319***
LA	0.192	0.200	RWC	0.073	0.136**
LM	0.112	0.143*	LNC	0.245	0.357***
SLA	0.081	0.06	LPC	0.127	0.196***
Chl a	0.064	0.169***	LKC	0.172	0.223*
Chl b	0.133	0.188**	LN/P	0.122	0.173
Morphology	0.180	0.176			
Physiology	0.140	0.226***			

## 4 Discussion

At altitudes ranging from 1375 to 1912 m, the responses of leaf functional traits in *S. superba* and *S. argentea* were similar, indicating strong environmental adaptability in both species. However, *S. argentea* demonstrated greater adaptability to high altitudes. At 1912 m, its chlorophyll content was significantly higher than that at 1375 and 1552 m. The effects of nitrogen and phosphorus limitation were observed only at altitudes above 1912 m. Furthermore, the RDPI_s_ of physiological traits in *S. argentea* were significantly higher than those of morphological traits, and the RDPI_s_ of most physiological traits were greater than those of *S. superba* .

### 4.1 Morphological Traits

By analyzing the ecological and physiological patterns of leaf traits along the altitude gradient, we can better understand and predict the responses of different plant species to climate change. Due to the high plasticity of leaves, changes in their functional traits may allow plants to adapt to other altitudes with unfavorable growth conditions (Guo et al. 2018). In this study, the SLA, LL, LW, LA, and LM values of the two species were markedly inversely proportional to the altitude, unlike the LT. In high‐altitude areas, plants typically reduce the transpiration rate as well as mitigate strong radiation‐induced damage as well as mechanical damage caused by low temperature and high wind speed by increasing the LT and reducing the LA and SLA (Wang et al. 2016; Thomas et al. 2020). This conservative strategy reduces energy loss (Midolo et al. 2019; Liu et al. 2020; Rixen et al. 2020; Zhang et al. 2024). Due to the close correlation between the SLA and leaf lifespan (Wright et al. 2004; Zhang et al. 2020), a decrease in the SLA with altitude may indicate a corresponding increase in the leaf lifespan of both species (Gratani et al. 2014). This study did not measure the leaf lifespan; however, the phenomenon of enhanced leaf lifespan in evergreen plants under colder environments has been widely documented (Wright et al. 2004; Zhang et al. 2010). This adaptability helps to maximize nutrient use efficiency by extending the average nitrogen retention time (Pfennigwerth et al. 2017). However, in low‐altitude areas, changes in leaf morphological traits may be related to resource competition and acquisition. Larger LA and LM values help plants gain an advantage in resource‐rich environments (Zhang et al. 2022). Additionally, increasing SLA in low‐ and medium‐altitude areas may be a strategy for species to adapt to altered conditions by maximizing their photosynthetic rate (Guo et al. 2018).

### 4.2 Physiological Traits

Photosynthetic pigments are responsible for light absorption and processing, which directly affect the photosynthetic capacity of plants. In this study, the Chl a, Chl b, and T Chl contents of the two species first increased and then decreased with increasing altitude, while Car content showed an increasing trend. These results corroborate those of Wingler et al. (2015) and Sharma et al. (2024). The reason for this change is that with the increase of altitude and the increase of light intensity, plants can absorb more short‐wave and long‐wave light, and participate in the increase of light energy transmission rate, thus promoting the biosynthesis of photosynthetic pigments (Razzak et al. 2017). However, the decrease in chlorophyll content at high altitudes may be explained as an effective protective mechanism, as high radiation, but low temperature, oxygen, and carbon dioxide concentrations can inhibit chlorophyll biosynthesis. Low temperature environments can also accelerate the decomposition of pigments, damaging the chloroplast structure, and decreasing chlorophyll concentrations. Therefore, the ability of plants to absorb short‐ and long‐wavelength light is weakened, and their participation in light energy transfer rate is reduced (Zhu and Yang 2015; Cui et al. 2018). Under such an environment, the amount of light absorbed by plants during photosynthesis exceeds the energy available to the photosystem, and the excess energy can damage the photosystem. Plants absorb ultraviolet radiation by upregulating Car. They can also employ heat dissipation to avoid the excessive absorption of ultraviolet radiation by chlorophyll, thereby reducing the damage caused by reactive oxygen species to the cell structure and further protecting the photosystem (Molina Montenegro et al. 2012; Alba and Munné‐Bosch 2020). In this study, the chlorophyll content of two species reached a peak at 1716 m, while at an altitude of 1912 m, the chlorophyll content of *S. argentea* decreased but remained significantly higher than that at 1375 and 1552 m. However, the chlorophyll content of *S. argentea* at 1912 m was only significantly higher than that at a low altitude of 1375 m. This shows that higher chlorophyll levels of *S. argentea* may offset reduced photosynthetic efficiency due to harsher environmental conditions and shorter growing seasons at higher altitudes (Bresson et al. 2009; Sharma et al. 2024). This difference reflects their precise environmental adaptation during long‐term evolution and also reveals their competitive advantages in varied ecological niches at different altitudes. *S. superba* performed better in the lower altitude area of 1552 m, whereas *S. argentea* had an advantage in the higher altitude areas. This effect may be related to genetic characteristics and niche differentiation.

Additioanlly, the SS, LNC, LPC, LKC, and LN/P values were directly proportional to the altitude. This trend is consistent with other studies (Bin et al. 2022). Under low temperatures, carbohydrates (especially SS) are often used as osmoprotectants, which can regulate the osmotic pressure of leaves to ensure normal water transport in plants, maintain the conformation of cell membranes and enzymes, and also serve as protective compounds against ROS in plants (Keunen et al. 2013; Díaz‐de‐Quijano et al. 2016). Bano and Fatima (2009) also noted that high‐altitude mountain herbs in the Hunza Valley of Pakistan have maximum sugar content. Most of the N in leaves exists in photosynthetic enzymes and chloroplasts; thus, the photosynthetic capacity is usually positively correlated with LNC (Mu and Chen 2021), as well as with the chlorophyll content of LNC. The variation in N and P content with altitude conforms to the “temperature plant physiology hypothesis,” which suggests that plants with higher N and P levels are more suitable for growing in areas with lower temperatures (Weih and Karlsson 2001; Zhang et al. 2019). Additionally, at high altitudes, in physiological terms, plants store the soil nutrients absorbed by their roots in their leaves to construct protective tissues, such as allocating higher N to insoluble protein fibers to enhance leaf cell wall toughness and thickness, thereby preventing excessive water loss caused by solar radiation (Song et al. 2012; Sharma et al. 2024). The increase in LPC and LKC is an adaptive strategy for plants to cope with low temperatures at higher altitudes. It can not only compensate for the decline in metabolic rate at low temperatures but also effectively regulate intracellular osmotic pressure and enhance cold and stress resistance (Li et al. 2015; Xu et al. 2018). Leaf N:P is an indicator reflecting the changes between the N and P limits. Altitude is significantly positively correlated with annual precipitation, and an increase in annual precipitation further drives an increase in the leaf N:P ratio (Chen et al. 2013). LN/*p* < 14 often indicates that plant growth is limited by N; N/*p* > 16 indicates that the plant is limited by P; and LN/P between 14 and 16 indicates that the plant is limited by N and P (Yan et al. 2017). In this study, the LN/P of *S. superba* was easily restricted by N at 1375–1550 m and was restricted by N and *p* > 1725 m. In contrast, *S. argentea* was restricted by N at 1375–1725 m but by N and P at > 1912 m. This N/P balance mechanism is common among high‐altitude plants, indicating that the nutrient limitation mechanisms and ecological strategies of the same species at different altitudes have different responses to climatic conditions (Wright et al. 2018), this variation reflects their vertical ecological niche differentiation, which is more conducive to the formation of dominant populations of *S. argentea* during resource competition at higher altitudes and promoting community stability. This study also found that RWC was directly proportional to altitude, which may be related to the slower evaporation rate of plants in high‐altitude areas. In addition, changes in leaf structure, such as a thickened cuticle and highly developed transport tissues, help to reduce water loss (Mangral et al. 2023; Sharma et al. 2024). Therefore, the adaptative mechanisms of plants at different altitudes do not exist in isolation but are the result of the synergistic influence of multiple physiological and biochemical processes. This comprehensive adaptation strategy not only helps plants survive in specific altitude ranges, but also provides the possibility of migration and diffusion across environmental gradients.

### 4.3 Comparison of Plasticity Indices

The magnitude of the phenotypic index reflects the extent of plant trait responses to heterogeneous environments. The plasticity of most physiological traits of *S. argentea* was markedly higher than that of *S. superba* , especially in Car, LNC, and SS—traits associated with nutrient and water acquisition, light protection strategies, and osmotic pressure regulation (Keunen et al. 2013; Zhu and Yang 2015; Ellsworth et al. 2022). This indicates that *S. argentea* possesses a higher capacity for physiological regulation and adaptability in response to changes in altitude. Compared with *S. superba* , *S. argentea* can enhance the accumulation of Car and SS at high altitudes. This not only allows it to dissipate excess light energy, absorb ultraviolet radiation, and reduce oxidative damage caused by reactive oxygen species but also to prevent cell dehydration by increasing cell fluid concentration, thus maintaining better physiological function in low‐temperature, high‐light environments (Díaz‐de‐Quijano et al. 2016; Bhat et al. 2016). Additionally, the high plasticity of LNC in *S. argentea* reflects its ability to effectively adjust photosynthetic capacity and chlorophyll content to adapt to varying light and temperature conditions across altitudes (Sharma et al. 2024). These adaptive strategies not only provide *S. argentea* with a competitive advantage in resource acquisition but also contribute to population stability and community structural complexity in high‐altitude environments. Therefore, the physiological plasticity of *S. argentea* is not merely a response to environmental gradients but a key factor in its successful reproduction and spread within ecosystems. However, aside from leaf biomass plasticity, which was significantly lower in *S. argentea* than in *S. superba* , the plasticity of other morphological traits in *S. argentea* was only slightly lower, with no significant differences between the two species. The plasticity of morphological traits is usually related to the allocation and utilization of resources under environmental factors such as light and temperature (Thomas et al. 2020). This is consistent with the conclusion of previous studies that the leaf morphology plasticity of shade‐tolerant trees is higher than that of shade‐intolerant trees (Niinemets and Valladares 2004; Beckert et al. 2014; Olguin et al. 2020). Physiological plasticity is related to colonization and growth in high‐light environments, whereas morphological plasticity is related to survival and growth in low‐light environments (Niinemets and Valladares 2004). Although *S. argentea* exhibits lower morphological plasticity in response to changes in altitude gradients, it is undoubtedly beneficial. The production and maintenance costs of physiological plasticity are considered lower than those of morphological plasticity; however, the benefits are significant because physiological changes occur in differentiated tissues that are imperceptible to the naked eye, whereas morphological plasticity is irreversible and involves the replacement of existing tissues by new tissues (Grime and Mackey 2002; Puglielli et al. 2017; Wang et al. 2023). Therefore, *S. argentea* is adaptable, selectively enhancing physiological plasticity and reducing morphological plasticity to gain survival advantages in higher altitude ecological niches in a more cost‐effective way, thereby avoiding the higher risk of improper response caused by prediction failure. Additionally, this phenomenon has been confirmed by the studies of most plant invasion scholars, in which invasive species exhibit enhanced physiological plasticity compared to noninvasive species, rather than morphological plasticity (Valladares et al. 2002; Wang et al. 2003; Feng et al. 2007; Hou et al. 2015). However, different perspectives suggest that plants with significant phenotypic plasticity in both leaf morphology and physiology are more environmentally adaptable (Brock et al. 2005; Richards et al. 2006; Bartoli et al. 2013). Generally, species with greater plasticity may better adapt to new or stressful environments, helping to expand their geographical or altitude range under varied environmental conditions (Molina Montenegro et al. 2012; Pfennigwerth et al. 2017). Our research also supports the view that *S. argentea* , which has a wider ecological niche, exhibits greater overall plasticity than *S. superba* . However, some plant invasion scholars have found no differences in plasticity between invasive and noninvasive species (Funk and Vitousek 2007; Palacio‐López and Gianoli 2011; Godoy et al. 2011).

## 5 Conclusion

Our research indicated that the response trends of the leaf traits of the two species to changes in altitude gradient were similar, but the adaptive potential of *S. argentea* was higher. When facing variations in altitude gradients, the RDPI_s_ of most physiological traits of *S. argentea* (wider ecological niche) were significantly higher than those of *S. superba* . In addition, the overall average RDPI_s_ of physiological traits of *S. argentea* were also significantly higher than its morphological traits, mainly reflected in the physiological traits (Car, SS, LNC) related to the species' nutrient and water acquisition, photoprotection strategies, and osmotic pressure regulation. This phenomenon reflects an efficient and economical adaptation strategy formed by *Schima argentea* in the long‐term process of adapting to the environment. These findings emphasize the importance of considering physiological plasticity when evaluating the adaptability of plants to environmental changes, especially in the case of widely distributed species. Future work may focus on exploring the mechanisms by which environmental factors affect the plasticity of plant traits and how these mechanisms shape the ecological niche and biodiversity patterns of plant populations.

## Author Contributions

**Deng Wang:** conceptualization (lead), data curation (lead), formal analysis (lead), funding acquisition (equal), investigation (equal), visualization (lead), writing – original draft (lead), writing – review and editing (lead). **Yeshe Wang:** funding acquisition (equal), investigation (equal), methodology (supporting), resources (supporting), supervision (lead). **Zhezhi Li:** investigation (supporting), methodology (supporting), writing – original draft (supporting), writing – review and editing (supporting). **Shu Wang:** investigation (supporting), methodology (supporting), writing – original draft (supporting), writing – review and editing (supporting). **Lijun Chen:** investigation (supporting), methodology (supporting), writing – original draft (supporting), writing – review and editing (supporting).

## Conflicts of Interest

The authors declare no conflicts of interest.

## Data Availability

Data are available via the Dryad Digital Repository: https://datadryad.org/dataset/doi:10.5061/dryad.rr4xgxdjr.
